# Chardonnay Grape Seed Flour Ameliorates Hepatic Steatosis and Insulin Resistance via Altered Hepatic Gene Expression for Oxidative Stress, Inflammation, and Lipid and Ceramide Synthesis in Diet-Induced Obese Mice

**DOI:** 10.1371/journal.pone.0167680

**Published:** 2016-12-15

**Authors:** Kun-Ho Seo, Glenn E. Bartley, Christina Tam, Hong-Seok Kim, Dong-Hyeon Kim, Jung-Whan Chon, Hyunsook Kim, Wallace Yokoyama

**Affiliations:** 1 College of Veterinary Medicine, Konkuk University, Hwayang-dong, Gwangjin-gu, Seoul, South Korea; 2 USDA, ARS, Albany, California, United States of America; 3 Department of Food and Nutrition, Hanyang University, Wangsimni-ro, Seongdong-gu, Seoul, South Korea; Universidad de Navarra, SPAIN

## Abstract

To identify differentially expressed hepatic genes contributing to the improvement of high-fat (HF) diet-induced hepatic steatosis and insulin resistance following supplementation of partially defatted flavonoid-rich Chardonnay grape seed flour (ChrSd), diet-induced obese (DIO) mice were fed HF diets containing either ChrSd or microcrystalline cellulose (MCC, control) for 5 weeks. The 2-h insulin area under the curve was significantly lowered by ChrSd, indicating that ChrSd improved insulin sensitivity. ChrSd intake also significantly reduced body weight gain, liver and adipose tissue weight, hepatic lipid content, and plasma low-density lipoprotein (LDL)-cholesterol, despite a significant increase in food intake. Exon microarray analysis of hepatic gene expression revealed down-regulation of genes related to triglyceride and ceramide synthesis, immune response, oxidative stress, and inflammation and upregulation of genes related to fatty acid oxidation, cholesterol, and bile acid synthesis. In conclusion, the effects of ChrSd supplementation in a HF diet on weight gain, insulin resistance, and progression of hepatic steatosis in DIO mice were associated with modulation of hepatic genes related to oxidative stress, inflammation, ceramide synthesis, and lipid and cholesterol metabolism.

## Introduction

Nonalcoholic fatty liver disease (NAFLD) is recognized as a significant public health problem. The prevalence of NAFLD is 20-30% of the general population of Western countries [1]. NAFLD ranges from steatosis (simple fatty liver) to nonalcoholic steatohepatitis (NASH), a condition that increases liver-related morbidity and mortality. Excessive hepatic lipid accumulation, oxidative and endoplasmic reticulum (ER) stress, inflammation, and insulin resistance are the major manifestations of progression of this disease. Although the etiology of NAFLD is not fully understood, the “two-hit hypothesis” is widely accepted [2]. In this hypothesis, excessive hepatic lipid accumulation is followed by increased oxidative stress and inflammation, resulting in liver damage.

Although NAFLD has no proven medical therapy [3], consumption of flavonoid-rich grapes and their products such as grape juice, wine, grape extracts, and purified compounds may be a favorable lifestyle modification that improves NAFLD by lowering hepatic lipid accumulation, inflammation, and/or oxidative stress [4]. Catechin inhibited lipid accumulation in HepG2 cells and mouse liver [5,6]. Supplementation with catechin-rich grape seed extract and catechin-rich Chardonnay grape seed flour (ChrSd) upregulated expression of genes related to fatty acid oxidation and downregulated genes related to fatty acid synthesis in liver [7–9], resulting in reduced hepatic steatosis. An extract from the pomace of the red American grape Norton induced anti-inflammatory effects in mice fed a high-fat (HF) diet and exhibited *in vitro* antioxidant activity [10]. An extract containing mainly procyanidin from Chardonnay grape seeds reduced oxidative stress markers and obesity in HF diet-induced obese (DIO) hamsters [11]. An extract from a pomace from five red and three wine grapes reduced obesity and insulin resistance and inhibited hepatic expression of genes involved in lipogenesis, gluconeogenesis, and inflammation in rats on a HF diet [12]. Intake of grape polyphenol extract for 8–9 weeks ameliorated high fructose-induced oxidative stress and insulin resistance in overweight, first-degree relatives of subjects with type 2 diabetes [13].

Most studies on the beneficial effects of grape seeds on NAFLD have been conducted using aqueous or alcoholic polyphenolic extracts of grape seeds [14–17]. Recently, the focus on the health benefits of flavonoids in grape products has expanded to wine grape seeds, a byproduct of the winemaking process. Whole grape seeds may contain significant amounts of unextractable phenolic compounds that contribute to their biological activity. Grape seeds contain two-thirds of the extractable flavonoids of grapes and have the highest concentrations of the most common flavonoids, flavan-3-ols (flavanols). The flavanols include catechin, epicatechin, their 3-O-gallates, and (epi)catechin dimers, oligomers, and polymers. Monomeric (epi)catechin is readily absorbed by the small intestine. Oligomers and polymers are not absorbed, but their phenolic acid metabolites produced by gut bacteria are absorbed.

We previously reported [9] that supplementation with partially defatted whole grape seed flour ameliorated hepatic steatosis, hypercholesterolemia, and obesity in hamsters. Hamsters fed a HF and hypercholesterolemic diet supplemented with seed flour produced from a byproduct of Chardonnay white wine had significantly lowered hepatic lipid contents, plasma concentrations of low-density lipoprotein (LDL), very low-density lipoprotein (VLDL), and total cholesterol. Their abdominal adipose tissue weight and body weight gain were reduced compared to those of hamsters on the control diet. These physiological changes were mediated by differential regulation of genes related to cholesterol, bile acid, and lipid metabolism in the liver and adipose tissues. In contrast, grape seed flours derived from red *Vitis vinifera* (Cabernet Sauvignon and Syrah) winemaking had little or no physiological effects. The beneficial health effects were correlated with the flavonoid content of the grape seed flour. This previous study in hamsters evaluated the expression of a limited number of selected genes since whole genome sequencing of the hamster has not been completed. In this study, to determine whether Chardonnay grape seed flour (ChrSd) supplementation modulates free radical scavenging, reducing oxidative stress, inflammation, and insulin resistance during the progression of HF-induced hepatic steatosis, we evaluated insulin tolerance and analyzed global gene expression by exon microarrays in livers from DIO mice supplemented with ChrSd.

## Materials and Methods

### Animals and Diets

Four-week-old male C57BL/6J mice were purchased from Jackson Laboratories (Sacramento, CA, USA) and housed individually in an environmentally controlled room (20−22°C, 60% relative humidity, 12-h alternating light/dark cycle). C57BL/6J mice show more susceptibility to HF diet-induced fatty liver disease than BALB/c mice [18]. Mice were acclimated and given *ad libitum* access to water and mouse chow diet (LabDiet 5015, PMI International, Redwood, CA, USA) for 1 week prior to initiation of experimental diets. Mice were weighed and randomized into two groups of 30 mice each. Mice were fed *ad libitum* with either mouse chow diet or a HF diet containing 17% of energy as protein, 37% as carbohydrate, and 47% as fat, with 0.1% cholesterol. After 5 weeks, mice were weighed, and DIO mice were identified as those having gained significantly more weight than the chow-fed mice. DIO mice were then randomized into two groups (n = 10 each) and fed *ad libitum* for 5 weeks with HF diets containing either 10% ChrSd (Sonomaceuticals, LLC/WholeVine Products, Santa Rosa, CA) or 5% microcrystalline cellulose (MCC, control diet; Dyets Inc., Bethlehem, PA) (Table 1). MCC, an insoluble fiber, has little effect on sterol metabolism [19]. Chardonnay grape pomace was obtained from coastal vineyards in Sonoma County, California, USA. Seeds from the 2010 vintage were dried using heated air (55-70°C) and separated from skins and stems. The residual press cake was milled to pass through an 85 mesh sieve, after oil had been pressed from the seed. Body weights were recorded weekly, and food intake was monitored twice per week. The study protocol, #P-04-02, was approved by the Animal Care and Use Committee, Western Regional Research Center, USDA, Albany, CA, USA.

**Table 1:** Diet Composition.

Ingredient (g/kg)	Con	ChrSd
Lard fat	225.0	225.0
Soybean oil	25.0	12.3
Cholesterol	0.8	0.8
MCC	52.6	18.6
Char seed*	0	100.0
Casein	200.0	182.5
Corn starch	145.6	109.8
Sucrose	300.0	300.0
dl Methionine	3.0	3.0
Choline bitartrate	3.0	3.0
Mineral mix	35.0	35.0
Vitamin mix	10.0	10.0
Total weight	1000.0	1000.0
Calories/kg	4444	4427
**Contents of Flavonoids in Chardonnay Grape Seed Flour**		
Polyphenol, mg/100 g		
Total flavonoids	12000	
Total catechins	1610	
Catechin	701	
Epicatechin	732	
Epigallocatechin	145	

Con (control diet containing 5% MCC, microcrystalline cellulose); ChrSd containing 10% Chardonnay grape seed flour;

*Contents of Chardonnay seed in g/100g were 16.1 protein, 11.7 fat, 59.7 carbohydrates, and 31.3 total dietary fiber; macronutrient composition was 47% of energy as fat, 16% as protein, and 37% as carbohydrate. Total dietary fiber contents were matched across diets.

### Plasma and Liver Collection

Mice were feed-deprived for 12 h and anesthetized by a vaporizer (VetEquip, Livermore, CA) with 4% isoflurane (Phoenix Pharmaceutical, St. Joseph, MO, USA) and 1L /min oxygen flow in an induction chamber. Recumbent animals were maintained at 2–4% isoflurane via nosecone, and blood was collected by cardiac puncture with syringes previously rinsed with potassium EDTA solution (15% w/v). Livers and epididymal adipose tissues were subsequently collected, weighed, and immediately frozen in liquid nitrogen for later analysis. Plasma was separated after centrifugation at 2,000 × *g* for 30 min at 4°C.

### Hepatic Total Lipid Content

After freeze-drying, powdered livers were weighed and mixed with 2 mL CHCl_3_/MeOH (2:1), sonicated for 5 min, and incubated overnight. Samples were centrifuged for 10 min at 1000 rpm, and supernatants were removed. Another 2 mL CHCl_3_/MeOH was added before sonicating and standing overnight for extraction. Solvent was removed from combined extracts under nitrogen, and total hepatic total lipid content was determined gravimetrically.

### Extractable Flavonoid Content

A 0.2 g sample was extracted with 20 mL methanol for 30 min with shaking, followed by sonication and centrifugation. Supernatants were subjected to high-performance liquid chromatography (HPLC) and total phenolic analysis. Total phenols and flavonoid compounds were analyzed using the Folin-Ciocalteu and standard HPLC methods, respectively, as previously described [20].

### Plasma Lipid Analysis

Plasma lipoprotein cholesterol was determined by size exclusion chromatography, as previously described [21]. HPLC was carried out using an Agilent 1100 HPLC chromatograph with a Superose 6HR HPLC column (Pharmacia LKB Biotechnology, Piscataway, NJ, USA) consisting of a mixing coil (1615–50 Bodman, Aston, PA, USA) in a temperature-controlled water jacket (Aura Industrials, Staten, NY, USA). A Hewlett-Packard HPLC pump (79851-A; Agilent Technologies, Palo Alto, CA, USA) was used to deliver cholesterol reagent (Roche Diagnostics, Indianapolis, IN, USA) at a flow rate of 0.2 mL/min. Bovine cholesterol lipoprotein standards were used to calibrate signals based on peak areas.

### Insulin Tolerance Test and Glucose Tolerance Test

For insulin tolerance test (ITT), after a 3-h fast, mice were administrated insulin intraperitoneally (0.5 U/kg body weight), and tail vein blood glucose levels were determined at 0, 30, and 60 min after insulin injection using a OneTouch Ultrameter (LifeScan Inc. Wayne, PA, USA). Glucose tolerance test (GTT) was performed after intraperitoneal administration of glucose (2 g/kg body weight). Blood glucose concentration was determined with tail-vein blood samplings at 0, 15, 30, 60, and 120 min after glucose injection using a OneTouch Ultrameter (LifeScan, Inc.).

### Gene Expression and Exon Microarray Analysis

Total liver RNA was extracted from three biological replicates within each group using a TRIzolplus RNA purification kit (Invitrogen, Life Technologies, Carlsbad, CA, USA). Total RNA quality was determined using a 2100 Bioanalyzer instrument and RNA 6000 Nano LabChip assay (Agilent Technologies, Palo Alto, CA, USA). Total RNA (10 μg) was used to synthesize one-cycle cDNA (first-strand and second-strand cDNA synthesis), followed by cleanup of double-stranded cDNA and biotin-labeled cRNA synthesis. Biotin-labeled cRNA was fragmented using One-Cycle Target Labeling and Control reagents (Affymetrix, Santa Clara, CA, USA). Fragmented cRNA samples were hybridized to Affymetrix GeneChip Mouse exon 1.0 ST arrays, expression and exon splicing arrays containing 1.2 million probe sets representing 80,000 genes. Hybridization signals were acquired and analyzed using a GeneChip Scanner 3000 High-Resolution Scanner (Affymetrix) and the Affymetrix GeneChip Operating Software. Analysis of gene expression and exon alternative splicing from microarray data were performed using GeneSpring GX version 11.0 program (Agilent Technologies, Santa Clara, CA). Gene expression was significant for changes |1.5|-fold and above. Splice index (SI) was defined as the log of the ratio of exon-level expression over gene-level expression. Fold change in SI value ±2 between treatment and control groups was considered differential splicing.

### Real-time PCR

Total RNA from livers was extracted using TRIzolplus RNA purification kit (Invitrogen, Life Technologies), and cDNA was synthesized using a GeneAmpRNA PCR kit (Applied Biosystems, Foster City, CA) per the manufacturer’s protocol. One microliter of diluted cDNA (1:10) was used in each real-time (RT)-PCR using SYBR Green Supermix (Bio-Rad, Hercules, CA, USA) with an Mx3000P instrument (Stratagene, Cedar Creek, TX, USA). Cycle conditions were as follows: 5 min at 95°C followed by 40 cycles at 94°C for 15 s, 55–60°C for 1 min, and 72°C for 30 s. Primers sequences are shown in Table 2 and in our [22,23] and other previous studies [24]. Primers were validated by PCR product sizes. No accumulation of nonspecific products or primer dimers was observed by gel electrophoresis of PCR products. Results were analyzed using software provided with the Stratagene Mx3000P QPCR system. Differences in mRNA expression were calculated after normalization to expression of 36B4 mRNA using the ΔΔCT method.

**Table 2:** Sequences of RT-PCR primers.

Gene	Product size (bp)	Primer Pair	5’ Primer sequence 3’
*Acsl3*	272	Forward	GAAGCTGCTATTTCAGCAAGTC
		Reverse	TAATATGAGGAATGGAGTTTG
*CFD*	282	Forward	CGACCTCATTCTTTTTAAGCTATC
		Reverse	CCGGAGTCTCCCCTGCAA
*Chi3l1*	288	Forward	TGACAGATACAGCAATGTGAACTAT
		Reverse	CACTGGTTGCCCTTGGTAG
*CHREBP*	204	Forward	ATGCGGGACATGTTTGATGACTA
		Reverse	ATTCAGGACAGTTGGCCGGAGAG
*GCK*	264	Forward	GTCGCAGGTGGAGAGCGACT
		Reverse	AACCGCTCCTTGAAGCTCG
*Gdf15*	124	Forward	ATACTCAGTCCAGAGGTGAGATTG
		Reverse	GCGTCAGCAGGAGCAGCG
*Plin4*	221	Forward	TGACAACTGAGGAACAAGC
		Reverse	CATGGTCATGTCTGTCATCTG
*RORC*	225	Forward	GAGCTCATCAGCTCCATATT
		Reverse	TTTGGGTGGCAGCTTGGCTA
*SpTlc3*	264	Forward	CCGCTAAAGTGTCTGCTTT
		Reverse	CTTCAAAGCTTGCTTCATTG
*SQLE*	214	Forward	GCTGCTATTTTCCAGGCCA
		Reverse	GGTGAGGAGACAATATTGAAA

### Hepatic ROS Level

ROS level in the liver was determined using the fluoresenct probe dichlorofluorescein (DCF) as previously described [25]. The stock solution of 2’,7’-Dichlorofluorescin diacetate (DCFDA, D6883, Sigma-Aldrich, St. Louis, MO) was prepared by dissolving DCFDA in 12.5 mM of ethanol and stored at -80°C until used. Approximately 20 mg of liver was homogenized in 0.5 ml HEPES buffered saline (140 mM NaCl, 5 mM KCl, 10 mM HEPES, 1 mM CaCl2, 1 mM MgCl2, 10 mM glucose), centrifuged at 1000 × g for 10 min. Homogenate containing 100 μg protein was pipetted into a black 96-well plate. The DCFDA was diluted to 125 μM immediately before use and pipetted into each well to a final concentration of 25 μM. The plate was placed on a shaker for 2 min and incubated at 37°C in the dark for 30 min. The fluorescence was read on a Wallac Victor 3 Multilabel Counter (PerkinElmer Inc., Waltham, MA) at excitation 485 nm/emission 530 nm at 0 and 50 min. The ROS level was expressed as the increased absorbance value between 0 and 50 min. Mean of fluorescence intensity was calculated from five mice per group.

### Western Blot Analysis

Frozen livers were sonicated in 50 mM Tris-HCl (pH 7.4), 250 mM NaCl, 5mM EDTA, 2mM Na_3_VO_4_, 1mM NaF, 20mM Na_4_P_2_O_7_, 0.02% NaN_3_, and 1% sodium dodecyl sulfate for 1 minute. Equal amounts of protein (100 mg), as measured with Bio-Rad Bradford protein assays and verified with Coomassie blue staining, were mixed with an equal volume of sample loading buffer, boiled for 5 minutes, and separated by electrophoresis on a 10% sodium dodecyl sulfate-polyacrylamide gel. Proteins were electrotransferred to polyvinylidene difluoride membranes and blocked with 5% non-fat dry milk in TBST (Tris-buffered saline, 0.05% Tween-20). Chi3l1, Sptlc3, and Aldh1a1 antibodies were from Abcam (Cambridge, MA) and Santa Cruz Biotechnology, Inc. (Dallas, TX). Antibodies were diluted and added to blots in blocking buffer. Blots were incubated overnight at 4°C, washed, and incubated with antimouse or antirabbit IgG secondary antibodies (Sigma-Aldrich, St. Louis, MO) labeled with horseradish peroxidase. Supersignal^®^ Westpico chemiluminescence substrate (Thermo Scientific, Waltham, MA) was used to visualize reaction complexes using X-ray film. Relative levels of proteins in tissues were determined using scanning laser densitometry (LKB ULTra San XL, Gelscan program), and mouse anti-β-actin (Sigma-Aldrich, St. Louis, MO) was used to normalize expression.

### Statistical Analysis

All data are expressed as mean ±SE. Analysis of variance was performed using the JMP7 statistical program (SAS Institute, Cary, NC, USA) to examine effects of treatment on plasma lipid level, body and tissue weights, total energy intake, and feed efficiency ratio. Significance was defined at *P* < 0.05. The Ingenuity Pathways Analysis tool (version 8.7, Ingenuity Systems Inc., Redwood City, CA, USA) was used to analyze exon microarray data determining biological mechanisms, pathways, and functions from differentially expressed genes. Right-tailed Fisher’s exact test was used to calculate *P* values, which represents the probability that the biological function of each dataset, biological function, and disease assigned to a particular network for each dataset and associations between the genes in a dataset and the corresponding canonical pathway were explained by chance.

## Results

### Body and Organ Weights, Energy Intake, and Metabolic Parameters

**Fig 1:**
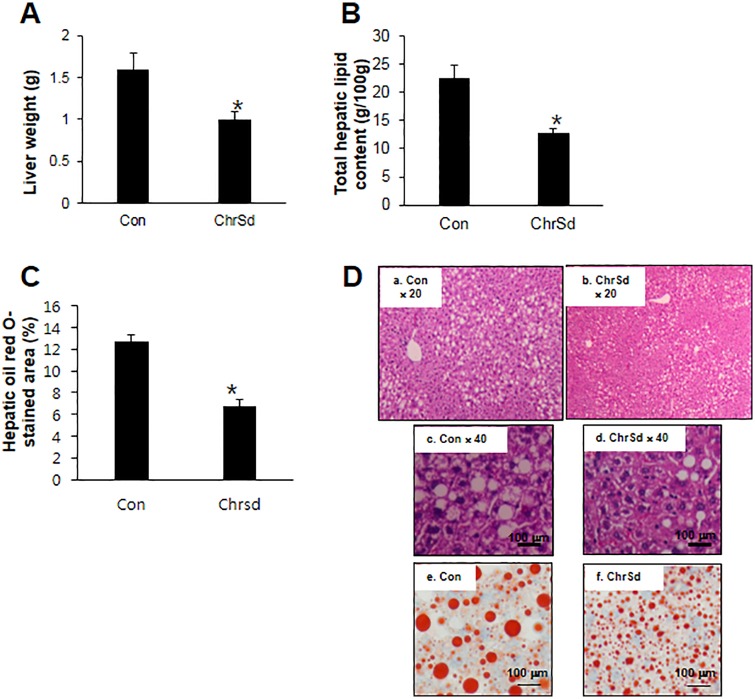
Effect of Chardonnay grape seed flour (ChrSd) on (A) liver weight, (B) total hepatic lipid content, (C) hepatic oil red O-stained area (%) and (D) H & E (a, b, c, & d) and oil red O (e & f) staining of liver tissues of male diet-induced obese mice (DIO) fed high-fat (HF) diets containing 5% microcrystalline cellulose (MCC, control) or 10% (w/w) ChrSd for 5 weeks. Data are expressed as mean ± SE; *n* = 8–10/group. *P < 0.05.

**Fig 2:**
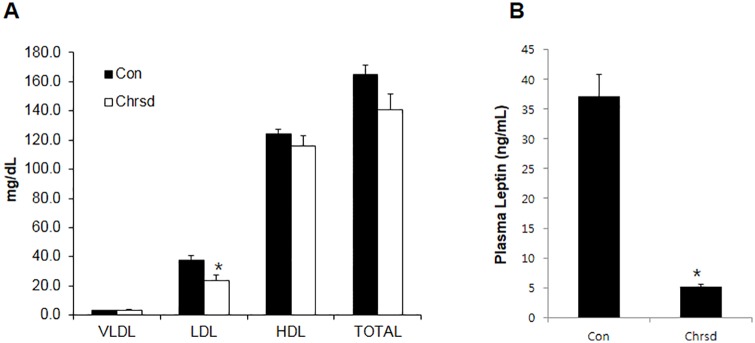
Effect of Chardonnay grape seed flour (ChrSd) on (A) plasma lipids and (B) leptin concentration. Male diet-induced obese mice (DIO) were fed high-fat (HF) diets containing 5% microcrystalline cellulose (MCC, control) or 10% (w/w) ChrSd for 5 weeks, and blood was collected in a food-deprived state. VLDL, very low-density lipoprotein; LDL, low-density lipoprotein; HDL, high-density lipoprotein. Data are expressed as mean ± SE; *n* = 8–10/group. *P < 0.05.

**Fig 3:**
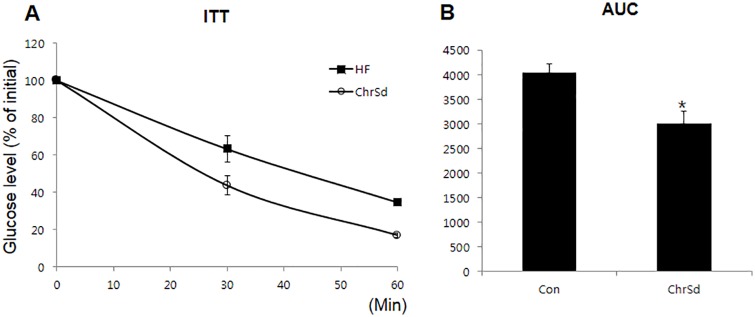
Insulin tolerance in obese mice fed a high-fat (HF) diet supplemented with either 5% microcrystalline cellulose (MCC, control) or 10% (w/w) Chardonnay grape seed flour (ChrSd) for 5 weeks. (A) Insulin tolerance tests (ITTs) were performed in the fasting state. (B) Area under the curve (AUC) values. Data are expressed as mean ± SE. *n* = 8–9/group. *P < 0.05.

ChrSd supplementation in a HF diet for 5 weeks significantly lowered body weight gain of DIO mice despite significant increases in daily food intake and total energy intake, resulting in 72% lower energy efficiency ratio in these mice (Table 3). Epididymal adipose tissue weight was 35% lower in DIO mice fed ChrSd compared with mice on the control diet (Table 3). The ChrSd supplemented diet significantly lowered liver weight by 38%, total hepatic lipid content by 43%, and hepatic oil red O-stained area by 47% compared to control diet (*P* < 0.05) (Fig 1A, 1B and 1C). Ratios of liver or adipose tissue weight to body weight were not affected by ChrSd supplementation (Con, 0.03 ± 0.003 and ChrSd, 0.03 ± 0.001). Significant reductions were seen in plasma LDL by 37% and leptin concentration by 86% when ChrSd diet was compared with control diet (*P <* 0.05) (Fig 2A and 2B). Fasting blood glucose concentration was significantly lower in mice on the ChrSd diet than on the control diet (206 ± 6.4 in control vs. 161 ± 9.4 mg/dL in ChrSd; *P* < 0.05). Dietary ChrSd supplementation resulted in markedly increased insulin response at 30 min and 60 min, lowering the area under the curve (AUC) during a 2-h insulin response (*P <* 0.05) (Fig 3A and 3B).

**Table 3:** Body and adipose tissue weights and energy intake in DIO mice fed MCC and ChrSd for 5 wk^1^.

	Con	ChrSd
Body weight gain (g)	2.4 ± 0.6	-2.0 ±0.7*
Initial body weight (g)	39.5 ± 1.0	37.1 ± 1.1
Final body weight (g)	41.9 ± 0.7	35.1 ± 1.1*
Total energy intake (Kcal)	676.0 ± 13.3	784.1 ± 20.1*
Daily food intake	3.0 ± 0.1	3.4 ± 0.1*
Feed efficiency ratio (g gain/g feed)	0.18 ± 0.00	-0.13 ± 0.00*
Epididymal adipose tissue weight (g)	2.0 ± 0.1	1.3 ± 0.1*
Ratio of epididymal adipose tissue weight to body weight	0.05 ±0.00	0.04 ± 0.00

^1^Values are mean ± SE, *n* = 10.

* Significant difference at *P* < 0.05.

### Microarray Analysis of Hepatic Gene Expression Profiles

Comprehensive expression of hepatic genes in DIO mice fed HF diets supplemented with either MCC or ChrSd flour was assessed by exon microarray analysis. A total of 205 genes, including one unknown gene, were differentially expressed in mice fed ChrSd compared to mice fed MCC (*P* < 0.05, fold change ≥ |1.5|) (Table 4). Among these genes, 102 were downregulated and 103 upregulated. Table 4 shows the selected genes differentially downregulated and upregulated by ChrSd, categorized by biological process. Chitinase-like 1 (*Chi3l1*; fold change, -1.5), encoding a protein involved in the activation of nuclear factor-kappaB (NF-κB)-induced kinase activity and involved in inflammation and tissue remodeling, was downregulated. A gene encoding an enzyme involved in corticosteroid biosynthesis (C21-steroid hormone biosynthesis, cytochrome P450, family 21, subfamily a polypeptide 1 (*Cyp21a1*; fold change, -1.6)) was downregulated. Expression of aquaporin 8 (*Aqp8*; fold change, -1.8), encoding a protein related to canalicular bile acid transport, was downregulated. Stearoyl-coenzyme A desaturase 1 (*Scd1*), a gene encoding a protein involved in monounsaturated fatty acid synthesis and monoacylglycerol O-acyltransferase 1 (*Mogat1*), involved in the diacylglycerol biosynthesis during triacylglycerol biosynthesis, were downregulated (fold change, -1.6 for both). Expression of odorant binding protein 2A (*Lcn13*), involved in glucose and lipid metabolism, was downregulated (fold change, -3.5). Genes involved in immune system processes, including complement factor D (*Cfd*; fold change, -2.4), cathepsin E (*Ctse*; fold change, -1.7), orosomucoid 2 (*Orm2*, fold change, -1.7), retinoic acid receptor-related orphan receptor gamma (*Rorc*; fold change -1.5), and toll-like receptor 5 (*Tlr5*; fold change, -2), were downregulated. A serine palmitoyltransferase, long-chain base subunit 3 (Sptlc3; fold change, -1.7), which is involved in sphingolipid metabolism, was downregulated. Expression of a lipid droplet-associated protein involved in the triglyceride metabolic process, perilipin 4 (*Plin4*; fold change, -3.0), was also downregulated in mice fed ChrSd compared to mice fed MCC. Interferon alpha 9 (*Ifna9*; fold change, 1.8), a gene encoding a protein related to host immune defense response, was upregulated. Expression of genes encoding cytochrome P450, family 7, subfamily b, polypeptide 1 (*Cyp7b1*; fold change, 1.6) and cytochrome P450, family 17, subfamily a, polypeptide 1 (*Cyp17a1*; fold change, 2.5), related to bile acid metabolism, was upregulated. Genes involved in cholesterol metabolism, including sterol 14-demethylase (*Cyp51*; fold change, 5.6), 3-hydroxy-3-methylglutaryl-coenzyme A reductase (*Hmgcr*; fold change, 2.5), hydroxysteroid (17-β) dehydrogenase 7 (*Hsd17b7*; fold change, 2.8), insulin induced gene 1 (*Insig1*; fold change, 1.8), sterol-C4-methyl oxidase-like (*Sc4mol*; fold change, 4.5), and leptin receptor (*Lepr*; fold change, 1.6) were upregulated. Expression of acyl-CoA synthetase long-chain family member 3 (*Acsl3*; fold change, 1.9), which is involved in fatty acid β-oxidation, was upregulated.

**Table 4:** Summary of selected genes showing significant ≥ |1.5|-fold hepatic modulation in mice fed a HF diet supplemented with ChrSd.

Biological Process (GO)^a)^	Gene Symbol	Name	Fold-Change	Gene ID
**Downregulated**				
Activation of nuclear factor-kappaB-inducing kinase activity	*Chi3l1*	Chitinase-like 1	-1.5	NM_007695
Acyl-CoA metabolic process	*Ces1d*	Carboxylesterase 1D	-1.6	NM_053200
Adrenergic receptor signaling pathway	*Adra1b*	Adrenergic receptor, alpha 1b	-1.6	ENSMUST00000067258
Aspartate transport	*Slc13a3*	Solute carrier family 13 (sodium-dependent dicarboxylate transporter), member 3	-1.7	ENSMUST00000029208
Brain-derived neurotrophic factor receptor signaling pathway	*Ntrk2*	Neurotrophic tyrosine kinase, receptor, type 2	-3.4	ENSMUST00000079828
C21-steroid hormone biosynthetic process	*Cyp21a1*	Cytochrome P450, family 21, subfamily a, polypeptide 1	-1.6	ENSMUST00000025223
Canalicular bile acid transport	*Aqp8*	Aquaporin 8	-1.8	NM_007474
Carbohydrate metabolic process	*Gck*	Glucokinase	-3.1	ENSMUST00000102920
Carbohydrate phosphorylation	*Pfkm*	Phosphofructokinase, muscle	-1.6	NM_001163487
Cell redox homeostasis	*Txn1*	Thioredoxin 1	-1.5	NM_011660
Cellular chloride ion homeostasis	*Ckb*	Creatine kinase, brain	-1.7	NM_021273
Cellular glucose homeostasis	*Gckr*	Glucokinase regulatory protein	-1.8	NM_144909
Cellular response to DNA damage stimulus	*Fen1*	Flap structure specific endonuclease 1	-2	ENSMUST00000156291
Cholesterol metabolic process	*Cyp46a1*	Cytochrome P450, family 46, subfamily a, polypeptide 1	-2.2	NM_010010
	*Vldlr*	Very low density lipoprotein receptor	-1.7	ENSMUST00000167487
Coenzyme A biosynthetic process	*Ppcdc*	Phosphopantothenoylcysteine decarboxylase	-1.5	NM_176831
Degradation of ketone body	*Bdh2*	3-hydroxybutyrate dehydrogenase, type 2	-1.6	NM_001172055
Fatty acid biosynthetic process	*Scd1*	Stearoyl-Coenzyme A desaturase 1	-1.6	NM_009127
Fatty acid metabolic process	*Acot11*	Acyl-Coenzyme A thioesterase 11	-2.2	NM_025590
Glucose homeostasis	*Mlxipl* (*ChREBP*)	MLX interacting protein-like	-1.6	NM_021455
Glucose and lipid metabolism	*Lcn13*	Odorant binding protein 2A	-3.5	ENSMUST00000077667
Glutamate biosynthetic process	*Prodh*	Proline dehydrogenase	-1.6	ENSMUST00000003620
Glutathione metabolic process	*Gstt3*	Glutathione S-transferase, theta 3	-1.7	NM_133994
G-protein coupled receptor signaling pathway	*Mc5r*	Melanocortin 5 receptor	-1.6	NM_013596
	*Tas2r104*	Taste receptor, type 2, member 104	-1.5	NM_207011
	*Vmn1r192*	Vomeronasal 1 receptor 192	-2.1	NM_145845
GTP catabolic process	*Rasl2-9*	RAS-like, family 2, locus 9	-2.2	NM_009028
Immune system process				
(Innate immune response)	*Cfd*	Complement factor D (adipsin)	-2.4	NM_013459
(Antigen processing and presentation of exogenous peptide antigen via MHC class II)	*Ctse*	Cathepsin E	-1.7	NM_007799
(Acute phase response)	*Orm2*	Orosomucoid 2	-1.7	NM_011016
(Regulation of gamma-delta T cell differentiation)	*Rorc*	Retinoic acid receptor-related orphan receptor gamma	-1.5	NM_011281
(Defense response to bacterium)	*Tlr5*	Toll-like receptor 5	-2	NM_016928
Methylation	*Hnmt*	Histamine N-methyltransferase	-1.5	NM_080462
Metabolic process	*Aldh1a1*	Aldehyde dehydrogenase 1 family member A1	-2.3	NM_11668
Oxidation-reduction process	*Cyp2b13*	Cytochrome P450, family 2, subfamily b, polypeptide 1	-3.4	NM_007813
	*Cyp2d40*	Cytochrome P450, family 2, subfamily d, polypeptide 40	-1.8	ENSMUST00000055721
	*Hao2*	Hydroxyacid oxidase 2	-1.7	NM_019545
Stress-responsive cytokine	*Gdf15*	Growth differentiation factor 15	-2	ENSMUST00000003808
Sphingolipid metabolic process (Ceramide de novo synthesis)	*Sptlc3*	Serine palmitoyltransferase, long chain base subunit 3	-1.7	ENSMUST00000110083
Triacylglycerol biosynthetic process	*Mogat1*	Monoacylglycerol O-acyltransferase 1	-1.6	NM_026713
Triglyceride metabolic process	*Plin4*	Perilipin 4	-3	NM_020568
**Upregulated**				
Adaptive immune response (host immune defense)	*Ifna9*	Interferon alpha 9	1.8	NM_010507
Asparagine biosynthetic process	*Asns*	Asparagine synthetase	2.8	ENSMUST00000031766
	*Got1*	Glutamate oxaloacetate transaminase 1, soluble	2	NM_010324
ATP hydrolysis coupled proton transport	*Atp6v0d2*	ATPase, H+ transporting, lysosomal V0 subunit D2	2.2	ENSMUST00000029900
Bile acid biosynthetic process	*Cyp7b1*	Cytochrome P450, family 7, subfamily b, polypeptide 1	1.6	NM_007825
	*Cyp17a1*	Cytochrome P450, family 17, subfamily a, polypeptide 1	2.5	NM_007809
Bone morphogenetic protein signaling pathway	*Id1*	Inhibitor of DNA binding 1	1.7	NM_010495
Calcium-mediated signaling	*Avpr1a*	Arginine vasopressin receptor 1A	2.1	NM_016847
Cell-cell junction organization	*Ocln*	Occludin	1.5	ENSMUST00000069756
Cholesterol biosynthetic process	*Cyp51*	Sterol 14-demethylase	5.6	NM_020010
	*Fdft1*	Farnesyl diphosphate farnesyl transferase 1	1.9	NM_010191
	*Hmgcr*	3-Hydroxy-3-methylglutaryl-Coenzyme A reductase	2.5	NM_008255
	*Hsd17b7*	Hydroxysteroid (17-beta) dehydrogenase 7	2.8	NM_010476
	*Insig1*	Insulin induced gene 1	1.8	NM_153526
	*Lss*	Lanosterol synthase	2.1	ENSMUST00000048678
	*Mvd*	Mevalonate (diphospho) decarboxylase	2.3	NR_028354
	*Mvk*	Mevalonate kinase	2.2	ENSMUST00000043760
	*Nsdhl*	NAD(P) dependent steroid dehydrogenase-like	2	NM_010941
	*Sc4mol*	Sterol-C4-methyl oxidase-like	4.5	ENSMUST00000034015
	*Tm7sf2*	Transmembrane 7 superfamily member 2	1.7	NM_028454
Cholesterol efflux	*Apom*	Apolipoprotein M	1.6	ENSMUST00000025249
Cholesterol metabolic process	*Lepr*	Leptin receptor	1.6	NM_146146
	*Pcsk9*	Proprotein convertase subtilisin/kexin type 9	1.9	NM_153565
	*Sqle*	Squalene epoxidase	13.5	NM_009270
Cytokine-mediated signaling pathway	*Il17rb*	Interleukin 17 receptor B	1.5	NM_019583
Fatty acid β-oxidation	*Acsl3*	Acyl-Coenzyme A synthetase long-chain family member 3	1.9	NM_028817
Glycine biosynthetic process, by transamination of glyoxylate	*Agxt*	Alanine-glyoxylate aminotransferase	1.5	NM_016702
Glycolytic process	*Aldoc*	Aldolase C, fructose-bisphosphate	2.2	ENSMUST00000017534
	*Pgk2*	Phosphoglycerate kinase 2	1.6	NM_031190
Lipid metabolic process	*Hmgcs1*	3-Hydroxy-3-methylglutaryl-Coenzyme A synthase 1	2.2	NM_145942
Lipid transport	*Stard4*	StAR-related lipid transfer (START) domain containing 4	1.9	ENSMUST00000025236
Proteinaceous extracellular matrix	*Mmp7*	Matrix metallopeptidase 7	1.5	ENSMUST00000018767
Regulation of insulin-like growth factor 1 receptor signaling pathway	*Igfbp2*	Insulin-like growth factor binding protein 2	2.3	NM_008342

^a)^ Genes were classified into biological process categories according to Gene Ontology (GO) Consortium classification.

**Fig 4:**
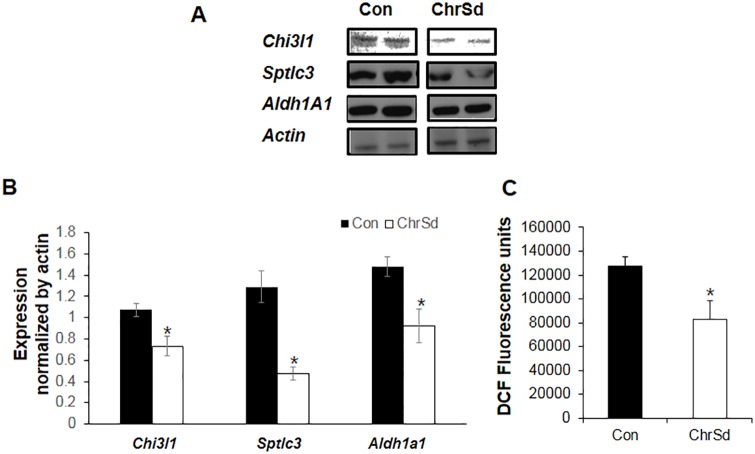
Reductions in Chi3l1, Sptlc3, and Aldh1a1 protein in livers from mice fed Chardonnay grape seed flour. (A) Western blots of Chi3l1, Sptlc3, and Aldh1a1 in protein extracts from livers of male, diet-induced obese mice (DIO) fed high-fat (HF) diets containing 5% microcrystalline cellulose (MCC, control) or 10% (w/w) ChrSd for 5 weeks. (B) Quantification of protein expression in (A). (C) DCF fluorescence intensity in the liver of male DIO mice fed HF diets containing 5% MCC or 10% ChrSd for 5 weeks. Data are expressed as mean ± SE; *n* = 3/group. *P < 0.05.

To confirm the microarray observations, 13 genes associated with cholesterol metabolism, fatty acid β-oxidation, glucose homeostasis, immune system, oxidative stress, and triglyceride metabolism were chosen for verification by RT-PCR. All genes showed expression patterns comparable to the microarray data (Table 5). To validate the microarray results at the protein level, we determined expression of Chi3l1, Sptlc3, and Aldh1a1, observing reductions of 3.1-fold for Chi3l1, 1.6-fold for Sptlc3, and 2.7-fold for Aldh1a1 in DIO mice fed ChrSd compared to mice on the control diet (Fig 4A and 4B). A consistent reduction was observed for all proteins when compared with the microarray data (Fig 4). DCF fluorescence intensity, indicator intracellular ROS level, in the liver of DIO mice fed ChrSd was significantly decreased by 35% compared with liver of DIO mice fed control diet (Fig 4C).

**Table 5:** RT-PCR validation of selected genes from microarray data.

Function	Gene	Fold	Microarray
Activation of nuclear factor-kappa β inducing kinase activity	*Chi3l1*	-5.5	-1.5
Carbohydrate metabolic process	*Gck*	-6.3	-3.1
Ceramide de novo synthesis	*Sptlc3*	-3.0	-1.7
Cholesterol synthesis	*Cyp51*	5.2	5.6
Cholesterol metabolic process	*Sqle*	24.4	13.5
Fatty acid β-oxidation	*Acsl3*	1.6	1.5
Glucose homeostasis	*Mlxipl (ChREBP)*	-2.6	-1.6
	*ChREBPα*	-3.2	-1.6
	*ChREBPβ*	-2.4	-1.6
Immune system	*Rorc*	-1.9	-1.5
	*Cfd*	-3.2	-2.4
Stress responsive cytokine	*Gdf15*	-3.1	-2.0
Triglyceride metabolic process	*Plin4*	-7.6	-1.5

Pathway analysis using the IPA System identified several biological functions and canonical gene pathways that were differentially regulated with ChrSd supplementation (S1 Table). When grouped by biological function, expression of genes related to lipid metabolism, hematological disease, and metabolic disease were significantly affected by ChrSd supplementation. For canonical pathways, ChrSd affected the cholesterol biosynthesis superpathway and the zymosterol biosynthetic pathway. Networks involving lipid metabolism and infectious disease were affected by ChrSd supplementation. Insig1, which regulates cholesterol concentration, was identified as a major regulator effect network (data not shown). Analysis of exon microarray data using the GeneSpring GX11.0 program identified 84 genes with SI > |2.0| in the presence of ChrSd supplementation (data not shown). Further analysis using RT-PCR is needed to confirm alternative splicing of these genes.

## Discussion

We previously showed that hypocholesterolemic and hypolipidemic effects of ChrSd supplementation of Golden Syrian hamsters on a HF and hypercholesterolemic diet are associated with altered hepatic expression of genes related to bile acid, cholesterol, and lipid metabolism [9]. In the current study, exon microarray analysis identified differentially expressed hepatic genes in DIO mice, providing novel biological insights into the effects of ChrSd supplementation on HF diet-induced hepatic steatosis. In addition to affecting expression of genes involved in cholesterol, bile acid, and lipid metabolism, dietary ChrSd supplementation significantly affected hepatic expression of genes involved in C21-steroid metabolism, immune system processes, inflammation, tissue remodeling, and lipid storage. ChrSd supplementation markedly upregulated hepatic expression of genes related to bile acid and cholesterol synthesis (*Cyp17a1*, *Cyp51*, *Hmgcr*, and *Insig1*) and fatty acid β-oxidation (*Acsl3*), whereas it significantly downregulated expression of genes related to fatty acid biosynthesis (*Scd1*, *Acot11*, and Mlxipl/carbohydrate responsive element-binding protein [*ChREBP*]), triacylglycerol biosynthesis (*Mogat1*), oxidative stress (growth differentiation factor 15, *Gdf15*), inflammatory and immune processes (*Cfd*, *Chi3l1*, *Ctse*, *Orm2*, *Rorc*, and *Tlr5*), ceramide biosynthesis (*Sptlc3*), and lipid storage (*Plin4*). These altered gene expression profiles in hepatic tissue were accompanied by significant reductions in plasma LDL-cholesterol concentration; liver, adipose, and body weight; feed efficiency ratio; and insulin resistance compared to the MCC supplemented control group. ChrSd diet attenuated HF-induced increase in hepatic ROS level, as shown by decreased DCF intensity in liver. Therefore, it appears that the decrease in expression of genes related to oxidative stress contributes to significant decreases of ROS level in livers of DIO mice following ChrSd diet. Notably, ChrSd supplementation lowered the hepatic expression of genes previously reported to be candidate genes for obesity, such as *Insig-1* [26], *lepR* [27], neurotrophic tyrosine kinase receptor type 2 (*Ntrk2*) [28], melanocortin 5 receptor (*Mc5r*) [29], and matrix metallopeptidase 7 (*Mmp7*) [30].

Based on previous studies showing that the antioxidant properties of flavonoids and hepatic have protective effects on inflammation and lipid accumulation [4,31–33], we hypothesized that supplementation with ChrSd containing high amounts of flavonoids would upregulate genes related to scavenging reactive oxygen species (ROS) and free radicals and downregulate genes related to oxidative stress, inflammation, and fatty acid biosynthesis, leading to improved HF-induced insulin resistance and NAFLD. The suggested mechanisms of the antioxidant activity of flavonoids are: 1) free radical scavenging and metal-chelating activities, 2) cell-to-cell signaling pathways, and 3) antioxidant enzyme gene expression [34]. The antioxidant activity of grape seed products has been monitored in *in vitro* [35,36]. The *in vivo* biological significance of *in vitro* activity is not clear because oligomers and larger flavonoids are poorly absorbed, while monomers are rapidly metabolized and cleared from the body. Nonetheless, previous studies reported that flavonoid-rich grape seed extract reduced oxidative stress markers in obese hamsters and human subjects [11,37,38]. In the present study, ChrSd supplementation down-regulated expression of stress responsive genes, *Gdf15* and *ChREBP*. Hepatic expression of *Gdf15*, a member of the transforming growth factor (TGF) β superfamily, is related to hepatic steatosis under ER stress [39]. Oxidative stress activates *ChREBP*, which transcriptionally modulates lipogenic and glycolytic genes [40], resulting in fatty liver. Taken together, these findings indicate that the flavonoid-rich ChrSd supplement potentiates antioxidant activity and reduces oxidative stress, resulting in transcriptional downregulation of ChREBP and Gdf15 and lipogenic genes (*Scd1*, *Acot11*, *Mogat1*) in the liver. Because we observed few changes in stress-related genes, canonical pathway analysis did not identify significant changes in genes associated with free radical- or ER stress-related signaling pathways in the livers of ChrSd-fed DIO mice. The explanations for the observed few changes in stress-related genes include the possibility that C57BL/6J mice fed a HF diet (47% of energy as fat) for 10 weeks may have already passed through the obesity-related ER stress response and entered the lipogenesis stage. This transition from ER stress to lipogenesis was shown in a previous study of ApoE3L mice on HF diets for 16 weeks; these animals exhibited an early phase hepatic response to the HF diet characterized by changes in genes linked to stress pathways, followed by a late phase involving genes related to lipid accumulation [41]. Additional explanations for the weaker free radical and ER-stress responses may be due to the shorter duration of the ChrSd supplementation phase of the study and the lower level of dietary fat compared to previous studies that showed an oxidative stress response [18,42,43]. C57BL/6J mice fed a HF diet (60% of energy as fat) developed hepatic steatosis and inflammation after 24 weeks [43]. Our study selected obesity-responsive mice by feeding C57BL/6J mice a HF diet for 5 weeks, then selecting DIO mice for treatment with HF diets supplemented with either ChrSd or MCC (control) for an additional 5 weeks.

Catechin-rich grape seed extract supplementation upregulated expression of genes related to fatty acid oxidation in mouse livers [8]. We also found that a catechin-rich ChrSd diet downregulated genes related to fatty acid synthesis (*Scd1*, *Acot11*, *Mogat1*) while upregulating genes related to fatty acid β-oxidation (*Acsl3*) compared to a control diet. As a result, the ChrSd diet lowered hepatic lipid content, contributing to the potential beneficial effect on hepatic steatosis. We note that our study did not determine activities of enzymes involved in fatty acid oxidation and synthesis. Changes in mRNA do not always measure pathway flux; however, previous studies have shown a linear relationship between fatty acid oxidation/synthesis activity and gene expression related to fatty acid metabolic pathways. For example, tea catechins upregulate both Acox1 mRNA and fatty acid activity in the liver [44].

Several studies have suggested that oxidative stress is important in the development of obesity-related complications such as insulin resistance and type 2 diabetes [38]. Consumption of red grape extract improved fructose-induced ER-stress and insulin sensitivity in healthy, overweight first-degree relatives of patients with type 2 diabetes [13]. Our study revealed that supplementation with flavonoid-rich ChrSd significantly downregulated expression of the hepatic stress responsive gene *Gdf15* and improved insulin sensitivity, as shown by 26% reduction in AUC during 2-h ITT compared to the control. ChrSd supplementation also significantly lowered fasting glucose concentration and AUC during a 2-h GTT (control AUC, 55,113 ± 2431; ChrSd AUC, 44,735 ± 2509, *P* < 0.01). Induction of *Gdf15* in response to oxidative stress and inflammation was increased in individuals with abdominal obesity, cardiovascular disease (CVD), and insulin resistance [45]. Therefore, downregulation of genes related to oxidative stress and inflammation by ChrSd supplementation might contribute to improved HF-induced insulin resistance. In addition, improved insulin sensitivity after ChrSd supplementation may be associated with the content of ceramides, lipid derivatives. Ceramide has been shown to inhibit insulin signaling and lipid accumulation in liver [46–48]. In our study, ChrSd supplementation downregulated expression of hepatic *Sptlc3*, a gene related to de novo ceramide synthesis. Flavonoids influence sphingolipid metabolism and normalize the elevated ceramide content of damaged liver cells [49]. Collectively, reductions in oxidative stress and ceramide biosynthesis may play an important role in the improvement of hepatic steatosis and insulin sensitivity in DIO mice supplemented with ChrSd.

Flavonoids have shown immunomodulatory and anti-inflammatory properties, although most studies were conducted *in vitro*, with few *in vivo* or human analyses [50]. In humans, consumption of 600 mg/day grape seed extract for 4 weeks improved insulin resistance and markers of inflammation such as blood C-reactive protein concentration in patients with type 2 diabetes at high risk for cardiovascular disease [51]. In our study, ChrSd supplementation downregulated hepatic expression of genes related to immune and inflammation processes: *Cfd*, *Chi3l1*, *Ctse*, *Orm2*, *Rorc*, *and Tlr5*. *Cfd* (*adipsin*) is a serine protease that is a host response factor in the removal of foreign antigens and pathogens [52]. The *Chi3l1* gene is involved in activation of NF-κB-induced kinase activity and inflammation [53]. *Ctse* is an intracellular aspartic proteinase highly expressed in immune-related cells such as macrophages. Its main role is in macrophage infiltration, adipogenesis, and hepatic steatosis [54]. *Orm2* is expressed in hepatocytes and adipocytes and secreted into plasma under metabolic and inflammatory stress as an acute phase reactant immunomodulator protein [55]. *Rorc* regulates Th17 cell differentiation, controlling the production of inflammatory cytokines. *Tlr5* is predominantly expressed in epithelial cells of the intestinal mucosa and has relatively low expression in liver and adipose tissue. A HF diet has been shown to increase *Tlr5* expression in mouse epididymal adipose tissue [56]. Expression is activated by bacterial flagellin proteins and triggers innate immune responses and NF-κB [57]. Although the biological significance of hepatic *Tlr5* downregulation is unclear, ChrSd supplementation may reduce the gut bacterial residence-derived inflammatory response to HF-induced stress. In our previous study, the composition of gut microbiota was altered by ChrSd supplementation, which was related to expression of intestinal fibroblast growth factor (*Fgf15*) and hepatic genes regulating cholesterol and lipid metabolism [58]. Collectively, these findings indicate that ChrSd supplementation prevents HF diet-induced inflammation of the liver by downregulating hepatic expression of genes related to immune and inflammatory pathways.

ChrSd supplementation upregulated hepatic leptin receptor expression by 1.5-fold and lowered plasma leptin concentration by 86% compared with controls. Leptin, a hormone produced by adipose tissue, regulates energy intake and expenditure and enhances fatty acid oxidation in liver and muscle [59]. In DIO mice, hepatic leptin receptor expression and enhanced plasma leptin concentration are reduced, suggesting that obesity induces hepatic insensitivity to leptin [60,61]. Thus, the ChrSd-associated upregulation of hepatic leptin receptor expression and downregulation of plasma leptin concentration suggest that improved hepatic leptin sensitivity could, in part, contribute to reduced hepatic steatosis.

Glucokinase (*Gck*) activation may induce fatty liver in rodents because *Gck* phosphorylates glucose to produce glucose-6-phosphate; this regulates hepatic glucose disposal and stimulates hepatic lipogenesis [62]. Recent studies have shown that *Gck* overexpression in the liver increases hepatic lipogenesis and circulating lipid concentrations [63,64]. Furthermore, hepatic *Gck* expression was associated with hepatic lipogenic gene expression and lipid content in human liver biopsies [65]. In our study, ChrSd supplementation downregulated hepatic expression of *Gck* and glucokinase regulatory protein (*Gckr*). This may also have contributed to reducing the HF-induced hepatic lipid content.

**Fig 5:**
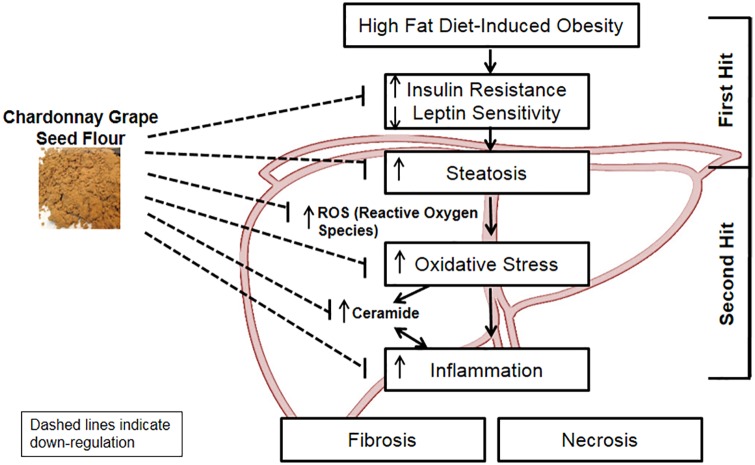
Proposed mechanisms by which flavonoid-rich Chardonnay grape seed flour (ChrSd) ameliorates high-fat (HF) diet-induced insulin resistance, hepatic steatosis, and nonalcoholic fatty liver disease (NAFLD). Supplementation with ChrSd lowers HF-induced insulin resistance and hepatic steatosis and enhances leptin sensitivity, followed by lowered oxidative stress and inflammation via reduction of ROS and ceramide synthesis. The result is possible amelioration of HF-induced progression of NAFLD. ROS, reactive oxygen species.

In summary, we demonstrated that flavonoid-rich ChrSd, a byproduct of winemaking, improved HF-induced hepatic steatosis, plasma lipid profiles, and insulin resistance. The health benefits of ChrSd may be due to high contents of flavonoids (Table 1). These improvements were associated with modulation of the hepatic expression of genes related to bile acid, cholesterol, and fatty acid metabolism; ceramide synthesis; oxidative stress; inflammation; and immune responses (Fig 5). Hepatic ROS level was significantly decreased by ChrSd diet. Pathways involved in lipid and cholesterol metabolism, and infectious and metabolic disease were differentially regulated by ChrSd. Future studies are required to confirm the proposed mechanisms (Fig 5). Our results suggest that consumption of flavonoid-rich ChrSd prevents NAFLD and other metabolic diseases by reducing oxidative stress and inflammation; modulating cholesterol, bile acid, and ceramide synthesis and lipid metabolism in the liver; and ameliorating insulin resistance.

## Supporting Information

S1 Table Top 10 biological functions and top five canonical and network pathways of genes significantly modulated by ChrSd. (DOC)
