# Supplementary material for: Chardonnay Grape Seed Flour Ameliorates Hepatic Steatosis and Insulin Resistance via Altered Hepatic Gene Expression for Oxidative Stress, Inflammation, and Lipid and Ceramide Synthesis in Diet-Induced Obese Mice
Source: PLoS One. 2016 Dec 15;11(12):e0167680. doi: 10.1371/journal.pone.0167680 (PMC5157984; doi:10.1371/journal.pone.0167680)
Supplement: S1 Table — (DOC) [file pone.0167680.s001.doc]

**Supporting information**

**S1 Table.** The top 10 biological functions and top 5 canonical and network pathways of genes significantly modulated by ChrSd

| Biological Function | *P* value | No. of genes differentially expressed | |
| --- | --- | --- | --- |
| Lipid Metabolism | 2.87 × 10^-10^‑3.11 × 10^-2^ | 48 | |
| Small Molecule Biochemistry | 2.87 × 10^-10^‑3.23 × 10^-2^ | 64 | |
| Vitamin and Mineral Metabolism | 2.87 × 10^-10^‑2.09 × 10^-2^ | 21 | |
| Molecular Transport | 2.52 × 10^-7^‑3.23 × 10^-2^ | 39 | |
| Cell Morphology | 6.92 × 10^-4^‑3.11 × 10^-2^ | 15 | |
| Hematological Disease | 5.48 × 10^-4^‑3.12 × 10^-2^ | 13 | |
| Metabolic Disease | 5.48 × 10^-4^‑3.11 × 10^-2^ | 25 | |
| Neurological Disease | 6.17 × 10^-4^‑3.11 × 10^-2^ | 35 | |
| Psychological Disorders | 6.17 × 10^-4^‑3.11 × 10^-2^ | 27 | |
| Dermatological Diseases and Conditions | 1.14 × 10^-3^‑1.05 × 10^-2^ | 18 | |
| Canonical Pathway | *P* value | Ratio^a)^ | |
| Superpathway of Cholesterol Biosynthesis | 1.81 × 10^-17^ | 12/87 (0.138) | |
| Cholesterol Biosynthesis I | 1.53 × 10^-13^ | 8/40 (0.2) | |
| Cholesterol Biosynthesis II (via 24,25-dihydrolanosterol) | 1.53 × 10^-13^ | 8/40 (0.2) | |
| Cholesterol Biosynthesis III (via Desmosterol) | 1.53 × 10^-13^ | 8/40 (0.2) | |
| Zymosterol Biosynthesis | 7.12 × 10^-10^ | 5/22 (0.227) | |
| Network Pathway | | Score^b)^ |  |
| Lipid Metabolism, Small Molecule Biochemistry, Vitamin and Mineral Metabolism | | 58 |  |
| Infectious Disease, Organismal Injury and Abnormalities, Renal and Urological Disease | | 36 |  |
| Protein Synthesis, Carbohydrate Metabolism, Lipid Metabolism | | 36 |  |
| Lipid Metabolism, Small Molecule Biochemistry, Vitamin and Mineral Metabolism | | 33 |  |
| Behavior, Neurological Disease, Endocrine System Development and Function | | 29 |  |

The functions and pathways that were most significant to the dataset were identified by Ingenuity Pathway Analysis (Ingenuity Systems).

a) Number of molecules (genes) that met the cut-off criteria, divided by the total number of molecules within a given pathway.

b) Likelihood of finding the focus molecules in a given pathway, expressed as the negative log of the *P* value.
